# Federated Learning Semantic Communication in UAV Systems: PPO-Based Joint Trajectory and Resource Allocation Optimization

**DOI:** 10.3390/s26020675

**Published:** 2026-01-20

**Authors:** Shuang Du, Yue Zhang, Zhen Tao, Han Li, Haibo Mei

**Affiliations:** 1 School of Aeronautics and Astronautics, University of Electronic Science and Technology of China, Chengdu 611731, China; sdu@uestc.edu.cn (S.D.); q1554788327@gmail.com (Z.T.); 2 Aircraft Swarm Intelligent Sensing and Cooperative Control Key Laboratory of Sichuan Province, Chengdu 611731, China; 3 Research Insitute of Electronic Science and Technology, University of Electronic Science and Technology of China, Chengdu 611731, China; yue.zhang@std.uestc.edu.cn; 4 Sichuan Keway Software Co., Ltd., Mianyang 621000, China; lihan@jezetek.cc; 5 Yangtze Delta Region Institute (Huzhou), University of Electronic Science and Technology, Huzhou 313001, China

**Keywords:** semantic communication, federated learning, deep learning, resource allocation, trajectory optimization, Unmanned Aerial Vehicle (UAV), Proximal Policy Optimization (PPO)

## Abstract

Semantic Communication (SC), driven by a deep learning (DL)-based “understand-before-transmit” paradigm, transmits lightweight semantic information (SI) instead of raw data. This approach significantly reduces data volume and communication overhead while maintaining performance, making it particularly suitable for UAV communications where the platform is constrained by size, weight, and power (SWAP) limitations. To alleviate the computational burden of semantic extraction (SE) on the UAV, this paper introduces federated learning (FL) as a distributed training framework. By establishing a collaborative architecture with edge users, computationally intensive tasks are offloaded to the edge devices, while the UAV serves as a central coordinator. We first demonstrate the feasibility of integrating FL into SC systems and then propose a novel solution based on Proximal Policy Optimization (PPO) to address the critical challenge of ensuring service fairness in UAV-assisted semantic communications. Specifically, we formulate a joint optimization problem that simultaneously designs the UAV’s flight trajectory and bandwidth allocation strategy. Experimental results validate that our FL-based training framework significantly reduces computational resource consumption, while the PPO-based algorithm approach effectively minimizes both energy consumption and task completion time while ensuring equitable quality-of-service (QoS) across all edge users.

## 1. Introduction

Nowadays, semantic communication (SC) is gradually attracting intensive research interests, and could potentially become a key technology of future 6G mobile networks [1]. SC was first proposed in the 1950s by R. Carnap et al. [2], and it is currently becoming a hot topic again, due to the emergence of sophisticated artificial intelligent (AI) technologies. Using Deep Learning (DL), the transmitter can easily achieve semantic extraction (SE) from raw data, like text [3], image [4], and speech [5,6]; and the receiver can carry out data inference/regeneration with regard to the extracted and transferred semantic information (SI) [7,8], considering the semantic effectiveness of the transmitted symbols. As a result, SC is the paradigm shift of Shannon’s Classical Information Theory (CIT), which adopts a “transmit-before-understanding” approach. Instead, SC leverages an “understand-before-transmit” strategy, thereby alleviating bandwidth pressure by reducing the amount of data to be exchanged and saving the communication cost. Further, thanks to the emergence of large language models (LLMs) [9], SC can be widely deployed with the help of artificial intelligence-generated content (AIGC) [10,11].

Due to its advantages, SC is particularly suitable for UAV communications. In traditional UAV communication, it follows CIT and jointly optimizes the UAV trajectory, resource allocation, air–ground communication scheduling, etc., to lead to a maximum UAV energy efficiency, while guaranteeing the QoS requirement of the air–ground communications [12,13,14] or the computation services offered by the UAV as a mobile edge computing server [15,16]. However, “Shannon’s trap” will be always the bottleneck of those traditional UAV communication systems. Furthermore, each UAV is constrained by its size, weight, and power (SWAP), which will severely limit the communication and computation functions in the air [17]. SC is very suitable to solve these issues in UAV systems. There have been a few works on UAV semantic communication, like [18,19]. Nevertheless, refs. [18,19] did not consider that each UAV may unable to handle the intensive computing on SE using DL methods, due to the SWAP constraints.

In [20,21], the authors all pointed out that distributed computing using edge intelligence is a promising technology to realize SC in systems with no consistent infrastructures, like UAV communications or IoT systems. Specifically, the authors in [20] proposed using federated learning (FL) [22] to solve the problem caused by limited computing power, energy constraint, and storage of the end devices during SC. On the contrary, without such distributed computing, those device constraints may result in long latency in training, updating, and knowledge sharing of the SE model, thereby degrading communication reliability. Ref. [23] introduced the design of the DL-based auction for computing resource allocation in SC-enabled Metaverse applications, where SC and edge computing are used as two disruptive solutions. However, there is no work so far considering the distributed edge intelligence for UAV semantic communications. In this paper, we apply federated learning to help UAV semantic communications.

Federated learning has been well-studied in UAV communications to improve the performance of UAVs in the computing and communication services they provide. Ref. [24] considered dynamic digital twin and federated learning for air–ground networks, where a UAV works as the aggregator and the collaborate digital twin model works as a trainer in the air. Ref. [25] developed an asynchronous federated learning (AFL) framework for multi-UAV-enabled networks, and designed a joint device selection, UAVs placement, and resource management algorithm to enhance the federated convergence speed and accuracy. Similarly, the works in [26,27,28] all considered using FL to enable UAV performances with joint optimization algorithms on various network settings. According to the best of our knowledge, no work considers has as yet considered FL used in UAV semantic communication. Overall, the contributions of this paper are as follows:

1. **Synergy of Federated Learning and Semantic Communication**: We propose a novel FL-based framework where edge users collaboratively train semantic communication models, with the UAV serving as the central server. This approach effectively distributes the computational load of semantic extraction, alleviating the processing burden on resource-constrained UAVs while maintaining data privacy.
2. **Distributed Semantic Extraction Paradigm**: To overcome UAVs’ computational limitations in deep learning-based semantic processing, we design a distributed training system that leverages edge intelligence to eliminate the computational bottlenecks inherent in deep learning-based semantic processing. This ensures that the UAV can maintain high-performance semantic communication without being overwhelmed by local processing requirements.
3. **Joint Trajectory and Resource Optimization via PPO**: Beyond just the learning architecture, we develop a PPO-based online optimization method. It uniquely integrates the dynamic flight trajectory with bandwidth allocation to satisfy the specific Quality-of-Service (QoS) and fairness requirements of semantic tasks, which are more complex than those in traditional bit-level transmission systems.
4. **Comprehensive Performance Evaluation**: Experimental results demonstrate that our FL-enabled semantic communication system achieves significant improvements in training efficiency and resource utilization, well addressing the computational constraints of UAVs. Meanwhile, the PPO-based trajectory optimization effectively minimizes energy consumption and task completion time while ensuring equitable QoS for edge users, fully meeting the design requirements of dynamic UAV-assisted semantic communication systems.

The remainder of this paper is organized as follows. Section 2 details the system model and outlines the associated problem formulation. In Section 3, we present the solution to the optimization problem. In Section 4, simulation results and analysis are presented. In Section 5, we provide conclusions and future work. *Other Notations*: In this paper, $C^{M\times N}$ denotes the set of M×N complex vectors. $R^{1\times N}$ denotes the array with *N* elements. diag{·} denotes the diagonalization operation. ${\left(\cdot \right)}^{T}$ denotes the transpose operation. E[·] denotes the expectation operation. ∥·∥ denotes the determinant operation.

## 2. System Model and Problem Formulation

**Figure 1:**
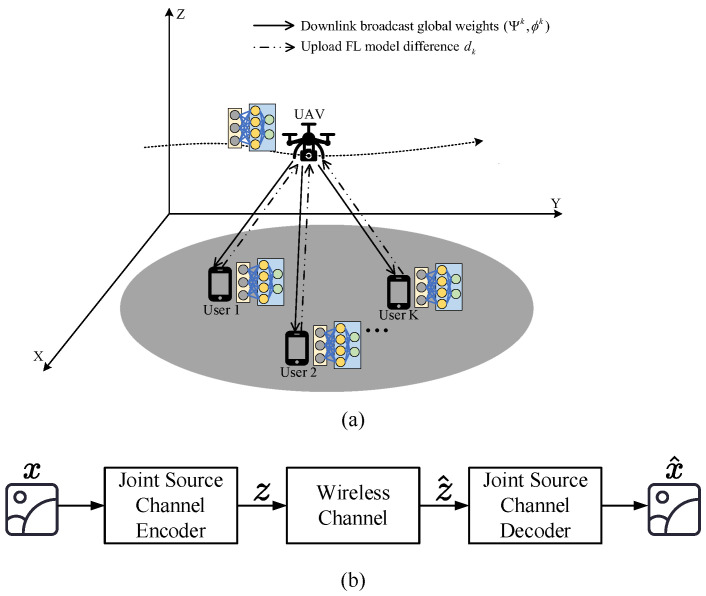
The system model on FL-enabled UAV semantic communications. (**a**) Architecture. (**b**) Block diagram of JSCC.

The architecture of the FL-enabled UAV semantic communication system is illustrated in Figure 1a. The UAV is denoted as *u*, and the users are denoted by k∈K={1,2,…,K}. Each user is located at a fixed position $p_{k}=\left(x_{k},y_{k},z_{k}\right)$, where $x_{k}$, $y_{k}$, and $z_{k}$ denote the 3D coordinates of the *k*-th user. To simplify the problem, we let $z_{k}$ = 0.

During the federated learning training phase, the UAV acts as a central server to aggregate model updates from all users for semantic communication. After the training is completed, the UAV will provide semantic communication services to the *K* users in the area.

We consider a discrete-time system where the time horizon is partitioned into *M* equal-length time slots, indexed by m∈M={1,2,…,M}. The duration of each time slot is $t_{s}$ seconds, representing the minimum time unit for UAV movement and communication operations. The position of the UAV at time slot *m* is represented by a two-dimensional coordinate vector $q_{m}=\left(x_{m},y_{m}\right)$, and to simplify the problem, we assume that the UAV maintains a constant altitude *H* throughout the process, i.e., $z_{m}=H$ for all *m*.

### 2.1. Movement Model for UAV

The operational airspace is modeled as a rectangular region with dimensions a×b, where *a* and *b* represent the width and length of the region, respectively. The position constraints of the UAV at the *m*-th time slot are given as follows:

(1a) $$\begin{array}{c} 0\leq x_{m},x_{k}\leq a,\forall m,k, \end{array}$$

(1b) $$\begin{array}{c} 0\leq y_{m},y_{k}\leq b,\forall m,k. \end{array}$$

Equations (1a) and (1b) ensure that both the UAV trajectory and user positions remain within the defined boundaries. The boundary values *a* and *b* define the spatial constraints of the mission area. In practical scenarios, these values are determined by the UAV’s flight endurance, the effective communication range of the semantic models, and local airspace regulations. For the purposes of this study, we set these parameters to reflect a typical urban or suburban deployment area, ensuring the UAV operates within a safe and manageable flight radius.

The kinematic model of the UAV is defined by the following equations. The velocity update equation is

(2) $$v_{m+1}=v_{m}+a_{m}t_{s},\forall m,$$

where $v_{m}$ denotes the horizontal velocity, $a_{m}$ denotes the acceleration during time slot $t_{s}$.

The position update equations are

(3a) $$\begin{array}{cc} x_{m+1} & =x_{m}+v_{m}t_{s},\forall m, \end{array}$$

(3b) $$\begin{array}{cc} y_{m+1} & =y_{m}+v_{m}t_{s},\forall m, \end{array}$$

where $\theta_{m}$ denotes the heading angle at time slot *m*, which controls the direction of movement in the 2D plane.

All parameters are subject to the following physical constraints:

(4a) $$\begin{array}{cc} 0 & \leq v_{m}\leq V_{\max},\forall m, \end{array}$$

(4b) $$\begin{array}{cc} 0 & \leq |a_{m}|\leq a_{\max},\forall m, \end{array}$$

where $V_{\max}$ denotes the maximum horizontal velocity of the UAV, and $a_{\max}$ is the maximum acceleration of the UAV.

### 2.2. Semantic Communication Model

To realize the SC between the UAV and users, a JSCC scheme is employed to integrate the SE and channel encoding/decoding into series of DL steps. With JSCC, each user can extract the semantic information of an image using the Convolutional Neural Network (CNN), and then the extracted SI will be jointly coded and transferred via noisy wireless channels. In this way, a user can transfer its local images to the UAV without intensive data volume.

The block diagram of JSCC is shown in Figure 1b. In the phase of training semantic communication based on federated learning, what we mainly consider is the semantic communication between users and UAVs. Let us assume the user takes the *n*-dimensional image as the *m*-th time slot input: $x_{m}$. To transfer $x_{m}$, the encoder of the user maps $x_{m}$ to a *k*-length vector of complex-valued channel input samples $z_{m}$. The encoding is carried out by means of a CNN representing deterministic encoding function $f_{\Psi }$: $R^{n}\to C^{k}$, with parameters Ψ. Following the encoding operation, the joint source-channel coded sequence $z_{m}$ is sent over the wireless channel by directly transmitting real and imaginary parts of the channel input samples over the I and Q components of the digital signal. The channel introduces corruption to the transmitted symbols, denoted by $\eta :C^{k}\to C^{k}$, and we model the transfer function as $\hat{z_{m}}=\eta \left(z_{m}\right)$. Afterwards, the decoder in the UAV maps the corrupted complex-valued signal $\hat{z_{m}}=\eta \left(z_{m}\right)\in C^{k}$ to an estimation of the original input $\hat{x_{m}}\in R^{n}$, using a decoding function $g_{\phi }:C^{k}\to R^{n}$ that is parameterized by the CNN with parameter set ϕ. Obviously, the decoder inverts the operations performed by the encoder to map the image features to an estimate $\hat{x_{m}}$ of the originally transmitted image. The encoding and decoding functions are designed jointly to minimize the average distortion between the original input image $x_{m}$ and its reconstruction $\hat{x_{m}}$ produced by the decoder:

(5) $$\left(\Psi^{*},\phi^{*}\right)=\underset{\Psi ,\phi }{\arg\min}L\left(\Psi ,\phi \right)$$

where $L\left(\Psi ,\phi \right)=\frac{1}{D}\sum_{x_{m}∼D}d\left(x_{m},\hat{x_{m}}\right)$ is the loss function of user; $d(x_{m},\hat{x_{m}}))$ is a given distortion measure, and *D* is the number of samples used in the model training of the user. Further details about JSCC can be found in [4].

In this paper we model the communication channel as a series of non-trainable layers in JSCC, and the correlated transfer function η can be denoted by

(6) $$\eta \left(z_{m}\right)=g_{m}z_{m}+N$$

where the vector $N\in C^{k}$ consists of independent identically distributed (i.i.d.) samples from a circularly symmetric complex Gaussian distribution function: $N∽\mathrm{CN}(0,\sigma^{2}I^{k})$, with average noise power $\sigma^{2}$; and $g_{m}$ is the channel gain under through the air–ground channel fading model between the UAV and the user, which can be represented as $g_{m}∽\mathrm{CN}\left(0,G_{m}\right)$ with normal random variable $G_{m}$.

In this study, we primarily leverage the mobility of the UAV to establish and maintain line-of-sight (LoS) links for point-to-point communication. While we acknowledge that shadowing effects may occur in complex environments, our proposed joint trajectory and resource allocation optimization is specifically designed to bypass obstacles, thereby maximizing the probability of LoS connectivity. Furthermore, given the relatively low operational speeds of the UAV in the considered scenarios, the impact of mobility-induced Doppler shifts on communication performance is marginal and thus omitted to maintain the tractability of the optimization problem. We consider $G_{m,k}$ is the close path-loss form between the UAV and *k*-th user at *m*-th time slot, it can be denoted as

(7) $$G_{m,k}=\left(20\log\left(\sqrt{{∥q_{m}-p_{k}∥}^{2}}\right)+\eta_{\mathrm{Los}}+C\right)$$

where $C=20\log(\frac{4\pi f_{c}}{c})$; $f_{c}$ is the carrier frequency (Hz) and *c* is the speed of light (m/s); $\eta_{\mathrm{LoS}}$ (in dB) is the loss corresponding to the LoS connection depending on the environment. Based on (7), the instantaneous signal to noise ratio (SNR) of the UAV’s link to the *k*-th user in time slot *m*, can be denoted by

(8) $${\mathrm{SNR}}_{m,k}=10\log_{10}\frac{PG_{m,k}}{B_{m,k}\sigma^{2}}$$

where *P* is the uplink transmit power of the UAV; and $B_{m,k}$ denotes the total available bandwidth between the UAV and *k*-th user in time slot *m* in Hertz (Hz).

For the FL training stage, we introduce the average path loss $G_{m}$, which quantifies the mean path loss from each UAV to each user. Mathematically, it is defined as

(9) $$G_{m}=\frac{1}{K}\sum_{k}G_{m,k}$$

The performance of the UAV SC is quantified in terms of PSNR, which measures the ratio between the maximum possible power of the signal and the power of the noise that corrupts the signal. The PSNR on the UAV sending the image to the user via SC is defined as

(10) $$\mathrm{PSNR}=10\log_{10}\frac{{\mathrm{MAX}}^{2}}{\mathrm{MSE}}$$

where $\mathrm{MSE}=\frac{1}{D}\sum_{x∼D}d^{2}\left(x,\hat{x}\right)$ is the mean squared-error between the reference image *x* and the reconstructed image $\hat{x}$; MAX is the maximum possible image pixels value of the image that is normally defined as the fixed value.

### 2.3. Federated Learning for UAV Semantic Communications

The effect of UAV SC is highly related to the encoder with CNN parameters Ψ and the decoder with CNN parameters ϕ. Therefore, it is vital to let each user train parameters Ψ and ϕ to reach high accuracy, i.e., minimizing L(Ψ,ϕ), respectively. In this paper, we thus establish a FL-based distributed architecture within users and UAVs, as shown in Figure 1a. Such FL architecture leads to effective SC between UAV and users, without costing too much in terms of the energy and computation resources of UAVs.

In the FL process, let Ψ,ϕ denote the global parameters of the JSCC model. The overall target of FL is to minimize the global loss function $\min_{\Psi ,\phi }L\left(\Psi ,\phi \right)=\sum_{k=1}^{K}\frac{D_{k}}{D}L_{k}\left(\Psi ,\phi \right)$ with $D=\sum_{k=1}^{K}D_{k}$, and broadcast the global parameters to each user for SC. In this way, each user can quickly obtain the global JSCC model parameters with high accuracy, instead of training those parameters on their own. This also saves the UAV from intensive energy consumption. To implement this FL process, we adopt the distributed approximate Newton (DANE) approach previously applied in FL over cellular wireless networks [29]. We then design an FL algorithm for UAV semantic communications, as shown in Algorithm 1.

Algorithm 1 goes to a loop with finite iterations to update the global model (Ψ,ϕ), and $\left(\Psi^{n},\phi^{n}\right)$ is the global FL model at a given iteration *n*. In one iteration, *k*-th user needs to solve the local optimization problem in parallel as

(11) $$\begin{array}{cc} \; & \min_{d_{n}^{k}}\;G_{k}\left(\Psi^{n},\phi^{n},d_{n}^{k}\right)≜L_{k}\left(\Psi^{n},\phi^{n},d_{n}^{k}\right)\\ \; & \;-{\left(\nabla L_{k}\left(\Psi^{n},\phi^{n}\right)-\xi \nabla L\left(\Psi^{n},\phi^{n}\right)\right)}^{T}d_{n}^{k} \end{array}$$

where ξ is a constant value; and $d_{n}^{k}$ is the difference between the global FL model and local FL model for *k*-th user, i.e., $\left(\Psi^{n},\phi^{n}\right)+d_{n}^{k}$ is the local FL model of user *k* at iteration *n*. Obviously, $G_{k}\left(\Psi^{n},\phi^{n},d_{n}^{k}\right)$ is the object function indicating how the local loss function is affected by the differences between the local and global FL models, with regard to the gradients of local and global loss functions. Steps 7 to 9 of Algorithm 1 solve the local optimization problem using the gradient method as

(12) $$d_{i+1,n}^{k}=d_{i,n}^{k}-\delta \nabla G_{k}\left(\Psi^{n},\phi^{n},d_{i,n}^{k}\right)$$

where δ is the step size; $d_{i,n}^{k}$ is the value of $d_{n}^{k}$ at the *i*-th local iteration with given global model $\Psi^{n},\phi^{n}$; and $\nabla G_{k}\left(\Psi^{n},\phi^{n},d_{i,n}^{k}\right)$ is the gradient of function $G_{k}\left(\Psi^{n},\phi^{n},d_{n}^{k}\right)$ at point $d_{n}^{k}=d_{i,n}^{k}$. With (12), one has $G_{k}\left(\Psi^{n},\phi^{n},d_{i-1,n}^{k}\right)\geq G_{k}\left(\Psi^{n},\phi^{n},d_{i,n}^{k}\right),\forall i$. Then, based on the predefined accuracy η, the local optimization in (11) can find solution ${d_{n}^{k}}^{*}$ ensuring

(13) $$\begin{array}{cc} \; & G_{k}\left(\Psi^{n},\phi^{n},d_{i,n}^{k}\right)-G_{k}\left(\Psi^{n},\phi^{n},{d_{n}^{k}}^{*}\right)\\ \; & \;\leq \eta \left(G_{k}\left(\Psi^{n},\phi^{n},d_{0,n}^{k}\right)-G_{k}\left(\Psi^{n},\phi^{n},{d_{n}^{k}}^{*}\right)\right) \end{array}$$

**Algorithm 1** FL Algorithm for UAV Semantic Communications**Require:** Global model $\Psi^{0},\phi^{0}$, local accuracy η, global accuracy ϵ, user set K **Ensure:** Optimized global model $\Psi^{n},\phi^{n}$  1:Initialize global model $\Psi^{0},\phi^{0}$, set global iteration n←0  2:**repeat**  3:     Each user k∈K computes $\nabla L_{k}\left(\Psi^{n},\phi^{n}\right)$ and sends to UAV  4:     UAV computes $\nabla L\left(\Psi^{n},\phi^{n}\right)=\frac{1}{\left|K\right|}\sum_{k=1}^{K}\nabla L_{k}\left(\Psi^{n},\phi^{n}\right)$  5:     UAV broadcasts $\nabla L(\Psi^{n},\phi^{n})$ to all users  6:     **for** each user k∈K in parallel **do**  7:         Initialize local iteration i←1, set $d_{0,n}^{k}\leftarrow 0$  8:         **repeat**  9:            Update: $d_{i+1,n}^{k}\leftarrow d_{i,n}^{k}-\delta \nabla G_{k}\left(\Psi^{n},\phi^{n},d_{i,n}^{k}\right)$10:            i←i+111:         **until** local accuracy η is obtained12:         Denote $d_{n}^{k}=d_{i,n}^{k}$13:         User *k* sends $d_{n}^{k}$ to UAV14:     **end for**15:     UAV computes: $\left(\Psi^{n+1},\phi^{n+1}\right)\leftarrow \left(\Psi^{n},\phi^{n}\right)+\frac{1}{K}\sum_{k=1}^{K}d_{n}^{k}$16:     n←n+117:**until** global accuracy ϵ is obtained18:**return**$\Psi^{n},\phi^{n}$

After each user finding ${d_{n}^{k}}^{*},\forall k$ with regard to accuracy η, the user will send ${d_{n}^{k}}^{*},\forall k$ to the UAV at step 10 of Algorithm 1. The UAV can update the global model in the current iteration at step 12. Afterwards, Algorithm 1 will go to another iteration with the updated global model $\left(\Psi^{n},\phi^{n}\right)$. Finally, the whole algorithm will stop and converge to a global accuracy ϵ. Then, the optimized global model $\left({\Psi^{n}}^{*},{\phi^{n}}^{*}\right)$ will be obtained, ensuring $\min_{\Psi ,\phi }L\left(\Psi ,\phi \right)=\sum_{k=1}^{K}\frac{D_{k}}{D}L_{k}\left(\Psi ,\phi \right)$. In other words, considering accuracy ϵ, the solution $\left({\Psi^{n}}^{*},{\phi^{n}}^{*}\right)$ is a point such that

(14) $$\begin{array}{cc} \; & L\left(\Psi^{n},\phi^{n}\right)-L\left({\Psi^{n}}^{*},{\phi^{n}}^{*}\right)\\ \; & \;\leq \epsilon \left(L\left(\Psi^{0},\phi^{0}\right)-L\left({\Psi^{n}}^{*},{\phi^{n}}^{*}\right)\right) \end{array}$$

With the local and global accuracies η and ϵ, we can find $I_{k}$ as the lower-bound of the number of local iterations of a UAV between step 7 and step 9. Based on L-Lipschitz and γ-strongly convex of the loss function $L_{u}$, $I_{k}$ can be defined as

(15) $$I_{k}=\upsilon \log_{2}\left(1/\eta \right),\forall k$$

where $\upsilon =\frac{2}{(2-L\delta )\delta \gamma }$; and δ<2/L; *L* and γ are determined by the loss function $L_{k}$, further explained in [29].

On the other hand, the lower-bound of the number of global iterations of Algorithm 1 $I_{g}$, can be defined as

(16) $$I_{g}=\frac{a}{1-\eta }$$

where $a=\frac{2L^{2}}{\gamma^{2}\xi }\ln\frac{1}{\epsilon }$, and $0<\xi \leq \frac{\gamma }{L}$. More details on defining the global and local lower bound of the FL algorithm can be found in [29].

We can observe from (15) and (16) that a high local accuracy η can save the global iterations, and a high global accuracy ϵ will lead to increased global iterations, and vice versa.

### 2.4. UAV Constraints on Semantic Communications

Due to the SWAP constraints, a UAV has limited energy and computation capacity. We first model the energy consumption of the UAV on propulsion, which consumes the major part of the UAV’s energy. According to [12], the propulsion energy cost in time slot *m* is

(17) $$\begin{array}{cc} \; & E_{m}^{r\text{-}\mathrm{uav}}=P_{0}\left(1+\frac{3{\left(v_{m}\right)}^{2}}{U_{\mathrm{tip}}^{2}}\right)+\frac{1}{2}d_{0}\rho sG{\left(v_{m}\right)}^{3}\\ \; & +P_{1}{\left(\sqrt{1+\frac{{\left(v_{m}\right)}^{4}}{4\upsilon_{0}^{4}}}-\frac{{\left(v_{m}\right)}^{2}}{2\upsilon_{0}^{2}}\right)}^{\frac{1}{2}} \end{array}$$

where $v_{m}$ is the horizontal velocity of the UAV along the time slot *m* and $t_{s}$ is the duration of each time slot. In addition, $P_{0}$ and $P_{1}$ are two defined constants, representing the blade profile power and induced power in hovering status, respectively. $U_{\mathrm{tip}}$ denotes the tip speed of the rotor blade; $v_{0}$ is known as the mean rotor induced velocity in hover; $d_{0}$ and *s* are the fuselage drag ratio and rotor solidity, respectively; and ρ and *G* denote the air density and rotor disk area, respectively. In this paper, assume the propulsion energy of the UAV is closely related to the horizontal velocity of the UAV in each path segment. For the purpose of exposition and more tractable analysis, we ignore the additional/fewer energy consumption caused by UAV acceleration/deceleration or rising/falling, which is reasonable for scenarios when the UAV maneuvering duration only takes a small portion of the total operation time.

Another part of the energy consumed by the UAV is for the model inferencing of JSCC. To support model inferencing, it is assumed that the on-board CPU frequency of the UAV is *F*, which is fixed. Then, the model inferencing energy consumption of UAV *u* is

(18) $$\begin{array}{c} E^{\mathrm{SE}}=\kappa CF^{2} \end{array}$$

where κ is the effective switched capacitance that depends on the chip architecture; *C* is the number of CPU cycles per sample in the UAV. To this end, the UAV *u* has the following overall energy constraint

(19) $$\begin{array}{c} \sum_{m=1}^{M}E_{m}\leq E_{\max} \end{array}$$

where $E_{m}=E^{r\text{-}\mathrm{uav}}+K\cdot E^{\mathrm{SE}}$, and $E_{\max}$ is the maximum allowed propulsion energy during the whole mission.

### 2.5. Problem Formulation

Given that this paper selects image transmission as a typical task for semantic communication, we assume that the UAV transmits an image with an original data volume of $D_{0}$ to a user. During the encoding process, the Joint Source-Channel Coding (JSCC) scheme performs compression on the image with a compression ratio of *C*. Consequently, the data volume of the encoded image can be simplified to $D_{0}C$. It is worth noting that the selection of *C* involves a fundamental trade-off: a smaller *C* enhances communication efficiency by reducing the required transmission time and energy, but it may also limit the restoration of fine-grained data features. In this study, *C* is configured to balance the transmission overhead with the quality of semantic reconstruction, ensuring the feasibility of real-time tasks in resource-constrained UAV environments.

In the communication link between the UAV and the user, let $B_{m,k}$ denote the channel bandwidth allocated by the UAV to the *k*-user in time slot *m*. Neglecting the overhead of link control signaling and the impact of channel error retransmission, the transmission time $\tau_{m,k}$ of the compressed image can be expressed as

(20) $$\tau_{m,k}=\frac{D_{0}C}{B_{m,k}\log_{2}\left(1+{\mathrm{SNR}}_{m,k}\right)}$$

We define a function, if the ${\mathrm{PSNR}}_{k}$ between the UAV and *k*-user is satisfied and the function value of user *k* in time slot *m* is set to 1 (indicating the user is successfully served); otherwise, it is set to 0.

(21) $$U_{k,m}=\left\{\begin{array}{cc} 1, & \mathrm{if}\;{\mathrm{PSNR}}_{k}\geq \zeta_{k}\;\mathrm{and}\;D_{0}\;\mathrm{is}\;\mathrm{transfered},\\ 0, & \mathrm{otherwise}, \end{array}\right.$$

where $\zeta_{k}$ represents the minimum PSNR requirement for user *k* and ${\mathrm{PSNR}}_{k}$ represents the PSNR between the UAV and *k*-user.

To ensure that the UAV provides as fair QoS as possible to each user throughout the entire flight procedure and avoid situations where individual users are continuously underserved, we define the service fairness constraint $f_{m}^{\mathrm{FI}}$ as the core metric for quantifying fairness at time slot *m* as follows:

(22) $$\begin{array}{c} f_{m}^{FI}=\frac{{\left(\sum_{k=1}^{K}\sum_{m^{\prime }=1}^{m}U_{k,m^{\prime }}\right)}^{2}}{K\sum_{k=1}^{K}{\left(\sum_{m^{\prime }=1}^{m}U_{k,m^{\prime }}\right)}^{2}} \end{array}$$

The more fair the service each user receives, the closer the coefficient approaches 1.

Our optimization objective is to minimize the combined cost of task completion time and energy consumption while ensuring fair QoS provision across all users and that the UAV returns back to the take-off point. Let $Q=\{q_{m}|\forall m\in M\}$ denote the UAV’s trajectory over *M* time slots, where $q_{m}=\left(x_{m},y_{m}\right)$ represents the 2D position at time slot *m*. Let $B=\{B_{m,k}|\forall m\in M,\forall k\in K\}$ denote the bandwidth allocation strategy. The joint optimization problem is formulated as

(23a) $$\begin{array}{cc} \;\;P:\max_{Q,B} & \;\frac{\sum_{m\in M}f_{m}^{\mathrm{FI}}}{\sum_{m\in M}t_{s}\times \sum_{m\in M}E_{m}} \end{array}$$

(23b) $$\begin{array}{cc} s.t.\;\; & \sum_{k=1}^{K}B_{m,k}\leq B_{\max},\forall m\in M \end{array}$$

(23c) $$\begin{array}{cc} \; & 0\leq x_{m}\leq a,\forall m\in M \end{array}$$

(23d) $$\begin{array}{cc} \; & 0\leq y_{m}\leq b,\forall m\in M \end{array}$$

(23e) $$\begin{array}{cc} \; & 0\leq \theta_{m}\leq 2\pi ,\forall m\in M \end{array}$$

(23f) $$\begin{array}{cc} \; & 0\leq v_{m}\leq V_{\max},\forall m\in M \end{array}$$

(23g) $$\begin{array}{cc} \; & q_{1}=q_{M} \end{array}$$

The optimization variables Q and B collectively determine service coverage, energy efficiency, and fairness performance, with their coupling effects directly influencing the achievable energy-delay product (EDP) under the given constraints.

## 3. Methodology

In the context of this framework, we consider the action space of the UAVs, including acceleration, pitch angle, and yaw angle. These actions will affect the flight trajectory of the UAVs, consequently impacting the PSNR quality provided by the UAVs to users, and influence the system’s environmental states. Furthermore, the previous states and actions collectively drive the agents to transition into new random states, eliciting instantaneous rewards. Consequently, the optimization problem is formulated as a Markov Decision Process (MDP) denoted by 〈S,A,P,R〉, where each UAV is considered as an agent. This agent interacts with a dynamic environment defined by a series of states $S=\{s_{m},m\in M\}$ and a series of actions $A=\{a_{m},m\in M\}$ to maximize long-term rewards $R=\{r_{m},m\in M\}$.

### 3.1. State Space

The state space should contain all relevant information about the environment. Therefore, we express the state of the UAV as consisting of

(24) $$\begin{array}{cc} s_{m}= & \{q_{m},v_{m},\theta_{m},\left\{u_{1},\Delta \zeta_{1},B_{m,1},N_{1}\right\},\\ \; & \ldots ,\{u_{K},\Delta \zeta_{K},B_{m,K},N_{K}\},\\ \; & E_{\mathrm{remain}},m\},\forall m\in M \end{array}$$

where $\zeta_{k}$ represents the user’s required PSNR, $u_{k}$ denotes the position of the *k*-th user, $\Delta \zeta_{k}$ represents the difference between the PSNR provided by the UAV and the PSNR required by the *k*-th user, $N_{k}$ denotes the cumulative number of completed communication tasks for the *k*-user, and $E_{\mathrm{remain}}$ denotes the remaining energy. Thus, the dimension of the state space is 4+4K+2. The transition of the remaining energy $E_{remain}$ is governed by the UAV’s power consumption. Specifically, the state update for energy is defined as $E_{remain,m+1}=E_{remain,m}-E_{m}t_{s}$, where $E_{m}$ is the total power consumption at time slot *m*, as formulated in Equations (17) and (18). This ensures that the agent can perceive its energy status and adjust its trajectory and resource allocation actions accordingly.

### 3.2. Action Space

The action space encompasses all possible measures that the UAV can take during the task. Therefore, we express the action of the UAV as consisting of

(25) $$a_{m}=\left\{a_{m},\nabla \theta_{m},B_{m,k}\right\},\forall m\in M,\forall k\in K$$

where $a_{m}$ denotes the normalized acceleration in time slot *m*, $\nabla \theta_{m}$ denotes the normalized yaw angle in time slot *m*, $\beta_{m}$ denotes the normalized pitch angle in time slot *m*, and $B_{m,k}$ denotes the allocated bandwidth ratio in time slot *m*. To reduce the dimensionality of the action space, we employ normalized values, which helps eliminate significant differences among features and promotes convergence. The normalized action can be represented as follows:

(26a) $$\begin{array}{cc} a_{m} & =\frac{a}{a_{\max}} \end{array}$$

(26b) $$\begin{array}{cc} \theta_{m} & =\frac{\theta }{2\pi } \end{array}$$

(26c) $$\begin{array}{cc} B_{m,k} & =\frac{B_{k}}{B_{\max}} \end{array}$$

where $a_{\max}$ represents the upper bound of acceleration, and a,θ, $B_{k}$ denote the actual values. Clearly, each vector value falls within a distinct range, specifically $a_{m}\in \left[0,1\right]$, $\theta_{m}$ and $B_{m,k}\in \left[0,1\right]$.

### 3.3. Reward

To ensure that the UAV successfully accomplishes its designated flight task while maintaining a logical closed-loop between energy observation and optimization, we have meticulously designed a comprehensive reward function. The agent receives an instantaneous reward *r* at each time slot, which guides the optimization of trajectory and resource allocation.

(27a) $$\begin{array}{cc} r_{1} & =\lambda_{1}\sum_{k=1}^{K}U_{k} \end{array}$$

(27b) $$\begin{array}{cc} r_{2} & =\lambda_{2}\frac{\sum_{m\in M}f_{m}^{\mathrm{FI}}}{\sum_{m\in M}t_{s}\times \sum_{m\in M}E_{m}} \end{array}$$

(27c) $$\begin{array}{cc} r_{3} & =-\lambda_{3},\;\mathrm{if}\;\left(23c\right),\;\left(23d\right)\;\mathrm{are}\;\mathrm{not}\;\mathrm{satisfied} \end{array}$$

(27d) $$\begin{array}{cc} r_{4} & =\frac{\lambda_{4}}{\sqrt{{\left(x_{m}-x_{1}\right)}^{2}+{\left(y_{m}-y_{1}\right)}^{2}}} \end{array}$$

To address the issue of sparse rewards, (27a) implements a progressive reward mechanism where the system accumulates gains as each user’s PSNR requirement is satisfied. Crucially, (27b) serves as the core efficiency reward that directly addresses the energy consumption observed in the state space. Since the remaining energy $E_{remain}$ in (24) transitions according to $E_{remain,m+1}=E_{remain,m}-E_{m}t_{s}$, the inclusion of $E_{m}$ in (27b) penalizes excessive energy expenditure resulting from high-speed maneuvers or intensive bandwidth allocation. This mechanism ensures that the agent perceives the “cost” of its actions on the energy state. Furthermore, (27c) enforces operational boundaries by imposing penalty terms when the UAV violates designated task areas, and (27d) provides a potential-based guidance to encourage the UAV to return toward the take-off point. The coefficients $\lambda_{1}$ to $\lambda_{4}$ represent the weights for each component. Finally, the total reward is expressed as

(28) $$r=r_{1}+r_{2}+r_{3}+r_{4}$$

### 3.4. PPO-Based Trajectory Optimization Algorithm

**Figure 2:**
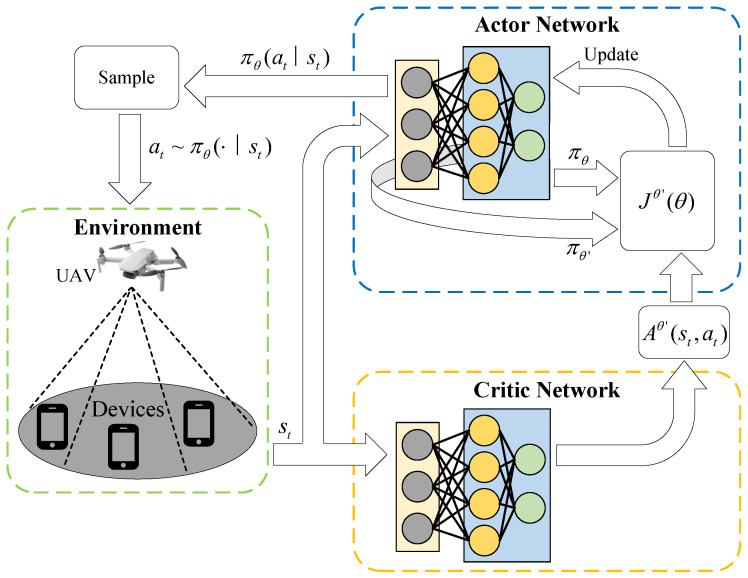
Architecture of the PPO-based trajectory optimization algorithm.

The procedural flow of the PPO-based algorithm is depicted in Figure 2, where the actor and critic networks jointly interact with the environment to derive the optimal computation offloading strategy. To reduce storage overhead, the proposed algorithm dispenses with a replay buffer and instead adopts an online training paradigm, in which learning is performed concurrently with execution. Details of the PPO-based trajectory optimization algorithm are shown in Algorithm 2.
**Algorithm 2** PPO-based UAV Trajectory Optimization Algorithm for Semantic Communications**Require:** Number of episodes N, actor update steps N, critic update steps I **Ensure:** Optimized policy π(a|s;θ)  1:Initialize actor parameters θ and critic parameters ϕ  2:**for** episode=1 to N **do**  3:      Collect trajectory $\tau ={\left\{\left(s_{t},a_{t},r_{t},done_{t},s_{t+1}\right)\right\}}_{t=1}^{T}$ using current policy $\pi (a|s;\theta^{N})$  4:      Set $\pi_{\mathrm{old}}\leftarrow \pi \left(a|s;\theta^{N}\right)$  5:      **for** each state $s_{t}\in \tau$ **do**  6:            Calculate advantage function ${\hat{A}}_{t}$  7:      **end for**  8:      **for** n=1 to N **do**              ▹ Actor update steps  9:            Calculate sampling ratio ${\hat{R}}_{t}^{n}=\frac{\pi (a_{t}|s_{t};\theta^{n})}{\pi (a_{t}|s_{t};\theta^{\mathrm{old}})}$10:            Calculate actor loss $L_{\pi }^{n}\left(\theta \right)$ using clipping function11:            Update actor parameters θ12:            Update policy: $\pi \left(a|s;\theta^{N}\right)\leftarrow \pi \left(a|s;\theta^{n}\right)$13:      **end for**14:      **for** j=1 to I **do**              ▹ Critic update steps15:            Calculate critic loss $L_{v}\left(\phi \right)$16:            Calculate gradient $\nabla L_{v}\left(\phi \right)$17:            Update critic parameters ϕ18:    **end for**19:**end for**20:**return** Optimized policy π(a|s;θ)

### 3.5. Computational Complexity Analysis

To evaluate the feasibility of Algorithm 1 for real-time UAV operations, we analyze its computational complexity. Let *T* denote the number of time slots per episode, while N and I represent the update steps for the actor and critic networks, respectively. The overall time complexity per episode is $O(T\cdot \left(NW_{a}+IW_{c}\right))$, where $W_{a}$ and $W_{c}$ are the computational weights of the actor and critic networks. In our implementation, both networks utilize a lightweight multi-layer perceptron (MLP) architecture with only two hidden layers. Such a streamlined structure ensures that the inference and training processes remain computationally tractable, allowing the UAV to perform trajectory planning and resource allocation within strict latency and energy constraints.

## 4. Numerical Results

In this section, we verify the feasibility and advantages of introducing federated learning into semantic communication. Subsequently, we validate the performance advantages of the reinforcement learning algorithm based on PPO in the joint optimization problem through simulations.

We consider that the users are randomly distributed in the targeted area, the targeted area (a,b,c) is set to (500m, 500m, 100m), and the flight altitude of the UAV is fixed at 50m. We consider that the user number in this area is 25 and their positions are randomly generated within this area. The UAV take-off point is set to (165m, 115m, 50m).

In the training stage, for the JSCC model, we select the CIFAR-10 dataset as the communication task and training data, with the compression ratio *C* set to 0.167. Due to constraints in hardware facilities and computational resources, three FL client nodes are configured in our experiments. This setup is conducive to ensuring stable algorithm convergence. Furthermore, three FL terminals are sufficient to provide a fundamental validation of the methodology’s feasibility and the effectiveness of the proposed joint optimization framework. Specifically, we set the global accuracy ϵ to 0.1 and the local accuracy η to 0.01, while the learning rate is configured as $1\times 10^{-5}$. For UAV parameters, the rotor blade tip speed $U_{\mathrm{tip}}$ is set to 100m/s; the mean rotor induced velocity in hover $v_{0}$ is 10m/s; the fuselage drag ratio $d_{0}$ is 0.1; the rotor solidity *s* is 0.1; the air density ρ is $1.225\;{\mathrm{kg}/m}^{3}$; the induced power in hovering status $P_{1}$ is 60W; the transmit power *P* is 10dBm; the blade profile power $P_{0}$ is 88W; the rotor disk area *G* is $0.28\;m^{2}$; the effective switched capacitance κ is $10^{-20}\;J/\mathrm{cycles}\cdot {\mathrm{Hz}}^{2}$; the number of CPU cycles per sample *C* is 600cycles; the on board CPU frequency *F* is 2.5GHz; the maximum horizontal velocity $V_{\max}$ is 50m/s; the maximum acceleration $a_{\max}$ is $10\;{m/s}^{2}$; the maximum energy $E_{\max}$ is 358,200 J; and the maximum bandwidth $B_{\max}$ is 10MHz. For environmental parameters, the carrier frequency $f_{c}$ is $2.4\times 10^{9}\;\mathrm{Hz}$; the noise density σ is −169dBm/Hz; the line-of-sight efficiency factor $\eta_{\mathrm{Los}}$ is 1; and the time slot is 1s.

In the optimize stage, we demonstrate the effectiveness of the proposed approach based on PPO in the reinforcement learning task through experiments. The experimental system is implemented using Python and PyTorch. For the actor and critic networks, we employ two fully connected hidden layers with 256 neurons each. The actor network is trained with a learning rate of $1\times 10^{-4}$, and the critic network is trained with a learning rate of $1\times 10^{-3}$. Adam optimizer is used to update the actor and critic networks. The reward weights are as follows: $\lambda_{1}$ is 5, $\lambda_{2}$ is 0.1, $\lambda_{3}$ is 10, $\lambda_{4}$ is 100. The reward weights $\lambda_{1}$ to $\lambda_{4}$ are treated as hyperparameters and are fine-tuned to ensure the stability of the training process and the convergence of the PPO algorithm. The selection of these values accounts for the relative scales of the different reward components and is informed by existing empirical studies in UAV-assisted communication [17], ensuring a balanced priority between service fairness and energy conservation.

After conducting multiple independent runs and performing statistical calculations, we obtained the following results.

**Figure 3:**
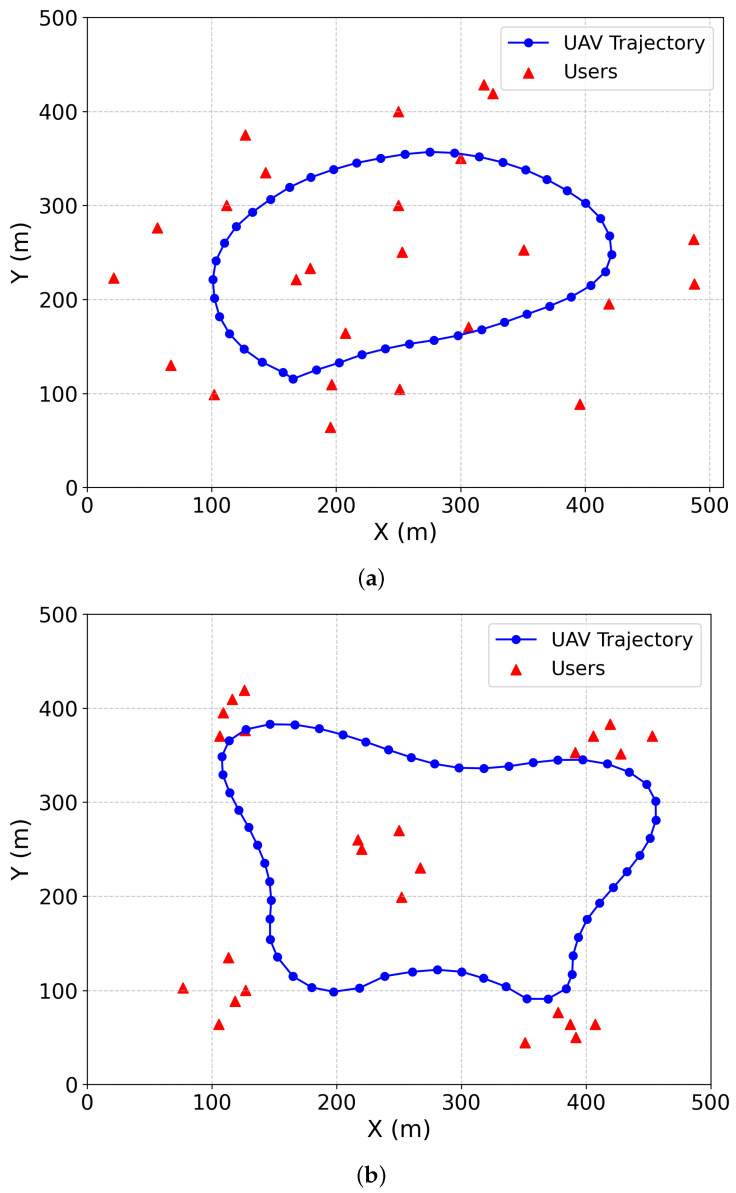
The trajectory of the UAV with 25 users. (**a**) Random distribution. (**b**) Clustered distribution.

According to Figure 3a,b, we can easily find that the trained UAV is observed to dynamically adjust its flight angle and acceleration in real time. Specifically, the UAV maintains its maximum speed to enhance task efficiency when the planned trajectory is relatively smooth; during turning maneuvers, however, it moderately reduces speed to ensure a stable transition.

To quantitatively evaluate the individual contributions of the FL framework and the PPO algorithm to the system performance, we conducted a comprehensive ablation study.

**Figure 4:**
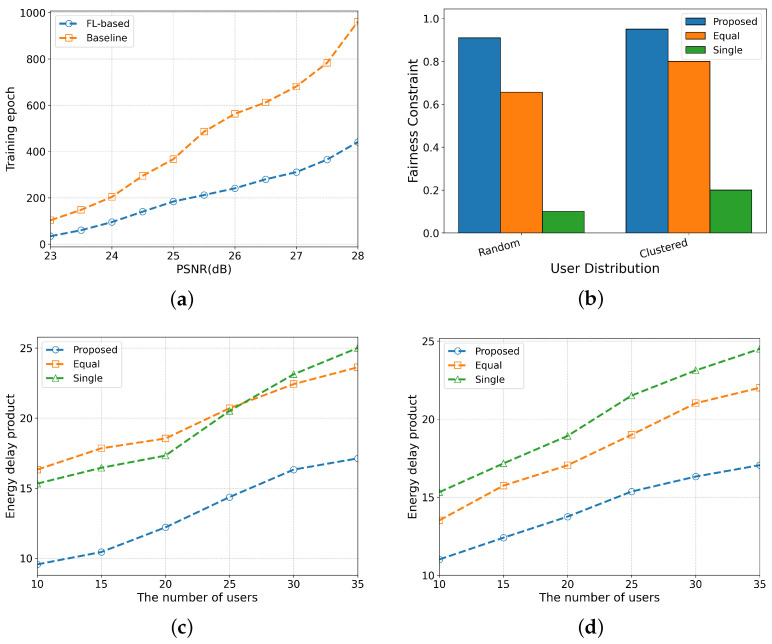
Results comparison of various algorithms and user distribution. (**a**) The training resource consumption of different methods. (**b**) User distribution impact on fairness constraint. (**c**) The EDP comparison in random distribution. (**d**) The EDP comparison in clustered distribution.

The training epoch requirement comparison between the FL-based training strategy and the traditional baseline method across varying PSNR values is shown in Figure 4a. As the figure shows, experimental results demonstrate that the FL-based method exhibits significant advantages in model training efficiency.

Figure 4b presents the fairness coefficients of the three methods under the scenarios of random and clustered user distributions: the (1) proposed method, which flexibly assigns bandwidth to each user based on actual needs; (2) the equal method, which distributes bandwidth evenly to all users per time slot; and the (3) single method, where only one user receives bandwidth resources per time slot.

Figure 4c presents the variation trend of the EDP over time, which shows a gradual upward trend with as time elapses, and the proposed method in this paper exhibits significant advantages: its EDP is consistently lower than that of other bandwidth allocation methods. The core reason for this advantage lies in the fact that the proposed method achieves precise resource trade-off through a dynamic bandwidth allocation strategy: for users at a further distance, a smaller bandwidth is allocated to meet their PSNR requirements; for users at a shorter distance, a larger bandwidth is allocated to reduce the service completion time. This differentiated bandwidth allocation mechanism enables services to be completed with lower energy consumption and in a shorter time. Eventually, the proposed method achieves the maximum average number of services completed. This performance advantage persists across different user densities, verifying the method’s robustness and practical value for UAV semantic communication systems.

## 5. Conclusions

To address the SWAP constraints in UAV-assisted systems, this paper proposes a training framework integrating FL and semantic communication. Simulations verify that this method can effectively reduce training resource overhead. On this basis, by formulating a multi-objective optimization problem and combining MDP modeling with the PPO algorithm for trajectory planning and dynamic bandwidth allocation, simulations also confirm its advantages in optimizing resource consumption. Moreover, the kinematically feasible trajectories generated by PPO outperform benchmark algorithms in both EDP and fairness.

While this study validates the effectiveness of the proposed FL-SC-PPO framework under a simplified system model, we acknowledge that real-world deployment entails more complex challenges. Consequently, our future work will extend this methodology to sophisticated environments by incorporating factors such as user mobility, varying UAV altitudes, and co-channel interference. Furthermore, while the current single-agent PPO-based framework demonstrates superior energy efficiency, exploring multi-agent reinforcement learning (MARL) and hybrid optimization methods remains a promising avenue for enhancing system scalability. These advanced architectures will be investigated in subsequent research to address more complex, coordinated multi-UAV semantic communication scenarios.

## Data Availability

This study uses the publicly available CIFAR-10 dataset, which can be accessed at https://www.cs.toronto.edu/~kriz/cifar.html (accessed on 14 December 2025). The synthetic data generated for UAV trajectory simulations and the implementation code for the proposed semantic communication algorithms are available from the corresponding author upon reasonable request.

## Abbreviations

The following abbreviations are used in this manuscript:

UAV	Unmanned Aerial Vehicle
FL	Federated Learning
SC	Semantic Communication
MEC	Mobile Edge Computing
LLM	Large Language Model
AIGC	AI-Generated Content
EDP	Energy-Delay Product
PPO	Proximal Policy Optimization
JSCC	Joint Source-Channel Coding
MDP	Markov Decision Process
PSNR	Peak Signal-to-Noise Ratio
SWaP	Size, Weight, and Power
